# A Formal Performance Evaluation Method for Customised Plug-and-Play Manufacturing Systems Using Coloured Petri Nets

**DOI:** 10.3390/s22207845

**Published:** 2022-10-15

**Authors:** Ge Wang, Di Li, Shiyong Wang, Minghao Cheng, Ziren Luo, Renshun Liu

**Affiliations:** 1 Shien-Ming Wu School of Intelligent Engineering, South China University of Technology, Guangzhou 511400, China; 2 School of Mechanical and Automotive Engineering, South China University of Technology, Guangzhou 510640, China

**Keywords:** cyber-physical production system, customised plug-and-play manufacturing, formal modelling, performance evaluation

## Abstract

Recent technological advancements and the evolution of industrial manufacturing paradigms have substantially increased the complexity of product-specific production systems. To reduce the time cost of modelling and verification and to enhance the degree of uniformity in the modelling process of system components, this article presents a componentised framework for domain modelling and performance analysis based on the concept of “multi-granularity and multi-view” for a production line of personalised and customised products, for plug-and-play manufacturing processes to involving a large number of model input parameters. The coloured Petri net tool is utilised as a simulation tool for mapping domain models to computational models for simulation and performance evaluation. This paper presents a method for setting the input parameters of a production system when using WIP, through-put and cycle time as metrics. The results of the performance analysis demonstrate the applicability of the proposed framework and provide direction for the production line’s layout design and scheduling strategy.

## 1. Introduction

The emerging paradigm of customised plug-and-play manufacturing (CPPM) is replacing mass-lot manufacturing and driving a new manufacturing trend. The shift in manufacturing has resulted in Industry 4.0, also known as the “fourth industrial revolution” [1]. CPS (cyber-physical systems) have been proposed in response to shifting industry and demand patterns. The cyber-physical production systems (CPPS) inherit the highly heterogeneous nature of the CPS and are utilised in the next generation of intelligent manufacturing modes [2]. The development of CPPS allows for customer participation in product design. Supported by CPPS, CPPM enables the production of various batch sizes and product types based on the direct needs of the customer [3]. However, the plug-and-play nature of CPPM has led to some new challenges in the product design phase. For instance, the frequency of formal validation is often inconsistent with the frequency of production plan modifications. This necessitates that formal verification in the system design phase meets two prerequisites: (1) Information about the system’s products and resources must be readily accessible, formalised and evaluated in a timely manner to meet functional properties such as system reliability. (2) Methods and tools exist to integrate formal models for functional verification and performance evaluation into a common modelling language to simplify model development and guarantee cost-effective operation.

Several advanced Industry 4.0 technologies and theories, such as Internet of Things (IoT) technology, cloud manufacturing, and service-oriented architecture, have been adopted to support CPPM. The widespread application of object linking for process control unified architecture (OPC UA) technology in manufacturing provides the technical basis for the design and validation of CPPS [4]. A CPPM should support formal verification based on real-time resource data to ensure the system model’s dependability even during runtime. Modelling experts also need to fully understand the interactions between production tasks and input parameters before modelling. Although the current CPPS architecture breaks down the traditional automation pyramid, the typical control behaviours remain, resulting in blocked point-to-point interactions between manufacturing resources and elements. The reason for this is that system asset information and data are designed for specific scenarios, and the information and data required during different lifecycle stages are often inconsistent. In other words, the problem of reused information has not been addressed. Componentisation [5] of systems is a technique for deconstructing systems into identifiable parts independently developed by programmers or modelling experts. Componentisation divides the system into distinct modules based on particular task scenarios, and each module and function is independent of the others. Model integration further complicates performance evaluation implementation due to the strict logical constraints imposed by functional verification of manufacturing systems. The components description of manufacturing systems is, therefore, a viable solution for enhancing information reuse and ensuring true cyber-physical domain integration.

The remainder of this article is organised as follows. In Section 2, a literature review of the involved work is introduced. Section 3 introduces the concept of CPPM componentised formal modelling and formal modelling methods. Section 4 provides an overview of the components and configuration of the CPPM system. The model development process is described in Section 5. The simulation’s experimental results are then presented in Section 6. The results are analysed, and the guidelines for determining the optimal input parameters are outlined. Section 7 explains the main conclusions and a discussion of future research.

## 2. Literature Review

Industry 4.0 manufacturing systems will face additional complexity introduced by the varying product requirements. This makes it difficult during the design phase to pre-configure an optimal solution for the wide range of execution variables during the design phase. Several techniques have been proposed for constructing dynamic formal verification models.

Some studies propose the static analysis and inspection of the product production process during the design phase using a rational and effective definition of the parameters, properties, and information of all components, resulting in a reusable formal model of the component [6]. López et al. [7] proposed a formal architecture that combines model-driven engineering with multi-intelligence (Agent) technology. The information model managed by the agent is used to customise the flexible manufacturing plant to individual requirements and to facilitate the dynamic addition of new products or resources. Mazak et al. [8] combined the advantages of Automation Markup Language (AutomationML) data modelling and model-driven engineering to enable the formal design and automated development of real-time data acquisition systems for manufacturing systems’ operational phases. Gradiar et al. [9] constructed formal models with mapping specifications and relationships for real-time resources using a data-driven methodology. As a result, by highly abstracting the various aspects of manufacturing system components to form system models for various purposes, manufacturing costs can be reduced and lead times for individual products shortened.

Furthermore, models for CPPM production lines often rely on uncertain input parameters [10], such as product arrival interval data, process consumption time data, reliability data (such as machine processing success), etc. These input parameters are simulated by the model to evaluate a data set representing the performance of the CPPM line. Consequently, evaluating the impact of the input parameters on the system’s performance is one of the most essential tasks during model simulation.

In early multilevel kanban-controlled manufacturing systems, the manufacturing system was modelled as a queuing network with a synchronisation mechanism [11]. Colledani et al. proposed the use of secondary decomposition techniques for the approximative analysis of the performance of computer-integrated manufacturing systems (CIMS) and complex machines [12]. Carlson et al. [13] applied a comparable strategy to an agile, synchronous manufacturing system. In [14], a performance analysis method for improving the index line of an asynchronous motor is proposed for a flexible manufacturing system with a limited shared buffer. Several researchers have proposed, for the purpose of improving system performance, a semantic data model-based virtual factory application that uses a semantic ontology approach to support the management and performance evaluation of flexible manufacturing systems, as described in [15,16]. However, the adaptation to the most recent technologies introduces intricate relationships between the various elements of the manufacturing system, which were rarely considered in the preceding work.

Undeniably, traditional formal theories and tools still play an important role in the modelling of manufacturing systems. Zimmermann et al. [17] proposed a modelling approach based on stochastic time-series coloured Petri nets, using dedicated coloured Petri nets to individually model the structure and production routes of manufacturing systems, and demonstrate typical failure and repair behaviour of automated machines modelled with stochastic Petri nets. Elwany et al. [18] developed a coloured Petri net model of a reliable six-station serial production line with limited buffer capacity. Through simulation experiments, the effects of station processing time variability, bottleneck location, buffer size and buffer space distribution on production capacity were investigated. Blaga et al. [19] developed Petri net models for a variety of manufacturing systems modelled and simulated with coloured Petri nets, such as production lines for the textile industry and assembly lines for the consumer goods industry. His research extends the application of generalized hybrid-timed Petri nets. Rak et al. [20] presented a programming tool based on timed coloured petri nets that supports the modelling and performance evaluation of system architectures for distributed system environments. The above studies have generally used CPN tools as a Petri nets modelling tool with good results. However, the traditional formal approach lacks real-time verification of the system, resulting in low efficiency.

Many organisations select Digital Enterprise Technology (DET) as a performance analysis tool [21]. The tools used in the solution are capable of solving most of the performance analysis problems encountered in the design phase of manufacturing systems. However, the commercial toolkit is a proprietary data structure that affects the consistency and interoperability of the production line’s components. In addition, such solutions are often prohibitively expensive to acquire and deploy, and they are rarely accessible to the average business. The application of formal methods in performance analysis addresses these issues and is widely adopted. Discrete event simulation based on formal MoCs such as Petri nets, Automata and Markov chains is an ideal tool for considering real-time constraints on manufacturing systems. A formal description and analysis method based on Process Specification Language (PSL) to generate parse logs based on finite automata theory is presented in [22]. Petri nets are capable of modelling and quantitatively and qualitatively analysing the dynamics of discrete event systems [23]. Consequently, it is most frequently employed to model and evaluate manufacturing systems. Kaid et al. [24] evaluated the resource utilisation and productivity of a flexible production line consisting of multiple machines using stochastic Petri nets. Petri nets have been extended in time to enable quantitative analysis of manufacturing system lead time in relation to throughput, as described in [25,26,27]. CPN tools, a commonly used modelling tool for the manufacturing domain, is the preferred choice for modelling production lines in new technological conditions, based on its good scalability [28].

However, there are still three challenges (CH) in a plug-and-play manufacturing system:

1. Customised manufacturing tasks necessitate frequent reallocation of resources and processes in response to changes in product orders. The production logic required by the reconfiguration depends on net properties as security, deadlock freedom in a preliminary design.
2. Due to the real-time data exchange of dynamic resources on the CPPM production line and a large number of model input parameters, it is challenging to accurately and objectively evaluate the impact of various production line factors such as product interval time, tray capacity and other production line inputs on production performance indicators.
3. How to integrate formal models for functional verification and performance evaluation into a common modelling language in order to simplify model development and guarantee cost-effective operation.

Therefore, this article proposes novel solutions to the mentioned challenges. The original works discussed are as follows:

- This study proposes a CPPM resource information model based on the concept of Industry 4.0 components. Based on the AutomationML format, the model constructs a multi-granular and multi-view modelling strategy that can dynamically describe the state of shop floor resources.
- A formal performance evaluation modelling framework for plug-and-play manufacturing systems is proposed based on coloured Petri Nets. Integration and unification of CPPM formal verification models and performance evaluation models are addressed.
- A series of simulation and analysis experiments was created to validate the proposed architecture, model, and analysis methods, as well as to propose a principle for determining optimal production input parameters and a design recommendation for line resources.

## 3. CPPS Componentised Formal Modelling Approach

This section provides an overview of the formal performance evaluation framework proposed for customised plug-and-play manufacturing, as well as the modelling techniques and performance evaluation techniques involved.

### 3.1. General Framework Overview

This article employs industrially mature techniques and tools to construct a reliable tool chain for modelling system components. Model Integrated Computing (MIC) [29] is a model-based design paradigm for the CPPM production line component utilising a domain-specific modelling language (DSML). The formal verification tool CPN tools are integrated by means of a model-to-model transformation. The solutions presented in this section are based on MIC-related methods and instruments.

**Figure 1:**
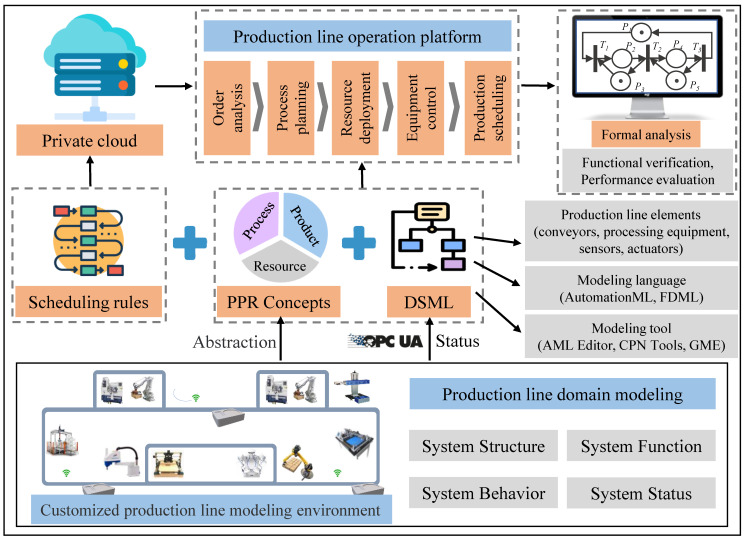
Framework of componentized formal method modelling.

The implementation of a model-driven framework based on the MIC context can be described in terms of the classic modelling structure proposed by an Object Management Group (OMG) [30], as shown in Figure 1. For the customised production line modelling environment, the four main aspects of the production line domain model in terms of system structure, system function, system behaviour, and system state are considered in order to realise the physical component system of the flow shop. Based on the definition of DSML, the production line elements are analysed and extracted from the classification “intelligent product–process–equipment–auxiliary sensing device operator” to determine the scope of application of DSML. AutomationML is used to construct the abstraction of the elements of the DSML semantic model in a set-theoretic approach to class inheritance, sharing, mutual exclusion, and dependency, as well as to assemble the associated constraint semantics into a domain formal modelling language. The detailed definition of the formal language is given in [25]. This article is based on the AutomationML data format and the PPR concept, as well as international standards for the meta-model. In the implementation process, the AutomationML editors for MIC Theory, CPN tools, and domain-specific modelling tools, such as GME, are utilised.

In practice, the domain model based on the customised production line modelling environment provides factory domain-specific modelling concepts that developers can use directly to achieve a straightforward description of the system in order to avoid inconsistencies between requirements and implementation. Scheduling rules defined in a constraint-based semantic rule language were introduced and deployed on a private cloud to facilitate real-time modelling and validation of production line jobs. The model supports the formal analysis of dynamic systems through the “model-data-driven” perception of the dynamic production environment, the self-organised scheduling of production resources, the free configuration of process parameters, and the adaptive adjustment of control strategies.

Using instances of the domain model as input, the model transformation is accomplished by defining mapping rules between the domain model and the formal model. Moreover, the customised production line layer employs OPC UA as a unified communication interface for the adaptation of resource entities. The AutomationML domain model was converted to the OPC UA information model and deployed to the control program’s development runtime tool. This approach enables the generation of code for actual production lines in order to facilitate the deployment of control node programmes for heterogeneous resource manufacturing units. This enhances the credibility of formal simulation results and the precision of performance analysis.

### 3.2. Design of the Cppm Production Line Component Concept

First, the concepts associated with CPPM components must be defined before they can be modelled. In general, CPPM component modelling is a complex process, and distinct component models must be guided by techniques and methodologies that are unique to each perspective or domain. There are three aspects of modelling that are problematic. The first challenge is the difficulty of integrating the multiple independently developed models required to construct the component’s informational part. The second requirement is that the form of physical component interconnection within CPPM components (including the definition of data and control flows) must be specified. Before the CPPM component can be deployed, the configuration parameters must be modified to correspond with production scenarios.

**Figure 2:**
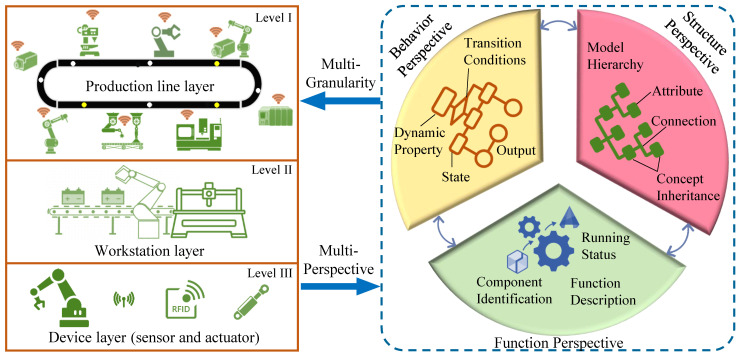
CPPM components multi-perspective and multi-granularity concept.

This section conceptually decouples line assets such as products, processes, and resources in CPPM production line components from different perspectives and at different structural, functional, and behavioural granularities. This allows the various types of components that support design and runtime to contain only the attributes and domain-specific knowledge of their respective domains. The multi-perspective concept indicates that the CPPM component has a structure–function–behaviour perspective, as shown in Figure 2. In particular, the structural perspective primarily describes the component’s static configuration, such as the component’s internal hierarchy and the structural topological relationship of the fine-grained component. In addition, the structural perspective describes the component’s static property parameters, such as geometrical parameters, material parameters, etc. The functional perspective describes the component’s capabilities and the collection of information accessible from the outside. This information includes the component’s global unique identifier, information about its function, and operational status. The global identification ensures global access to the various production line components during the design and operation phases. The component function information describes the range of capabilities that the component is capable of achieving. The operational status information is used to indicate the current operational status of a CPPM component and to determine the stage at which the component can perform the required functions for a specific production scenario. The behavioural perspective defines the state and state transfer relationships within components and the behavioural paradigms for semantic-based formal computation, optimisation, and negotiation/combination.

Multi-granularity indicates that the refinement and combinatorial relationships of CPPM components are relatively fine. CPPM components can be divided by granularity into atomic components and composite components. Components of varying granularity have nested relationships; for instance, atomic components can be combined to form composite components, and composite components can be combined to form composite components with higher granularity. As depicted in Figure 2, system resource components can be subdivided into various granularities, including production lines, workstations, and equipment. Several different devices can be combined into workstations, and several different workstations can be combined into production lines. The same principle can be applied to the granularity of other types of components, including products, processes, and other assets. In addition, the use of multi-perspective methods to abstract CPPM components with varying granularity can result in a unified model description of multiple perspectives.

### 3.3. Model Transformation Method

In the model validation phase, the conversion of the domain model to the CPN model needs to be implemented and the simulation validated in CPN tools. This article develops an application tool based on the model transformation algorithm on an integrated development platform (General Modeling Environment, GME) for the automatic generation of formal models. GME is a configurable toolset with a modular and expandable architecture that provides an extended interface for third-party tool integration in order to implement the transformation of AutomationML domain models to formal models. The CPN tools utilise the CPN ML programming language, which is derived from the functional programming language Standard ML. A model transformation plug-in for CPN tools has been developed in the GME environment. Using this plug-in, the CPN model for formal verification and performance analysis can be derived from the AutomationML-based component model of a production line system. A detailed model development is presented in Section 5. The model interpreter *CPNExporter* is developed based on the interface provided by GME to complete the conversion of the domain model to the CPN model. The development of the model interpreter CPNExporter relates to the model interpreter of the GME export class.

The algorithm in Algorithm 1 represents the pseudo-code of the *CPNExporter* interpreter model program. First, the C++ classes generated from the AML metamodel and CPN model’s XSD files under GME are imported into the *CPNExporter* model transformation framework. The planning and scheduling information from the GME domain model is then updated to the CPN model within GME, and the CPN template file is updated based on this CPN model. The updated CPN file adds token information representing the product.


**Algorithm 1: Generate CPN Model**


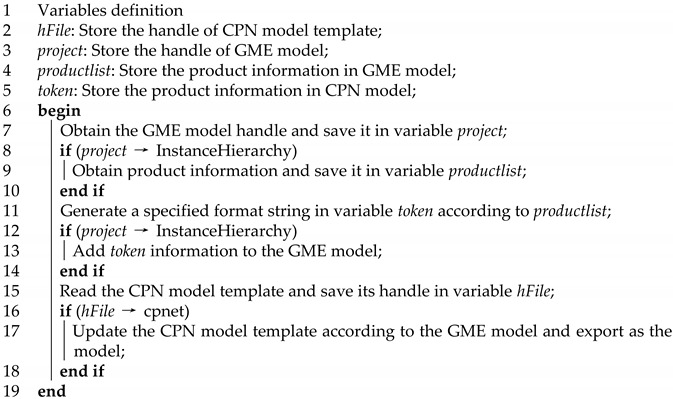



## 4. Composition and System Configuration of the CPPM Production Line

**Figure 3:**
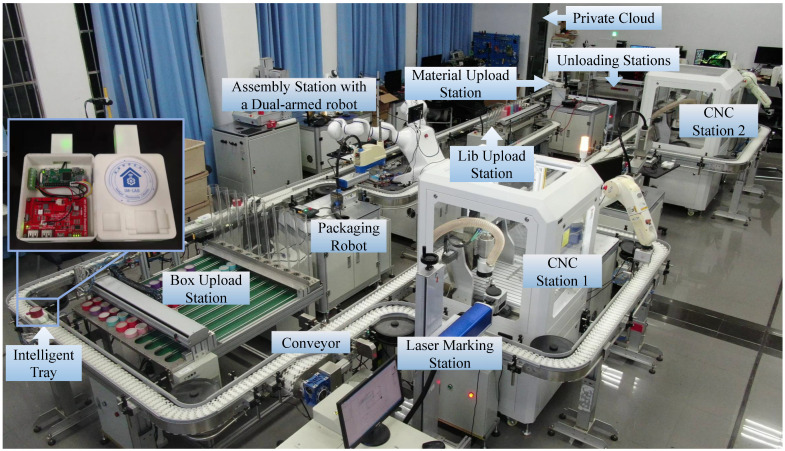
Intelligent factory experiment platform system configuration.

As shown in Figure 3, this section introduces a case utilising the experimental platform of the Industry 4.0 intelligent factory production line. This experimental platform utilises an intelligent control system to schedule and coordinates the relevant equipment, allowing for the automatic production of three products, each of which contains multiple models, on a mixed line.

### 4.1. Hardware Distribution and Network Composition

The experimental platform’s hardware and network consist of the following components: (1) a number of intelligent pallets capable of real-time interaction with the private cloud; (2) nine workstations offering different processes and six intelligent variable-speed conveyors; (3) a private cloud server comprising five rack-mounted servers; (4) the communication facility consists of a system comprising three switches, one router, and operational control tools.

All relevant equipment and workstations are equipped with resource adapters. The resource adapters connect the heterogeneous devices to the private cloud via a wired or wireless network. When the order system receives an order, the private cloud establishes machine handling procedures and process and resource utilisation strategies. Communication facilities disseminate these strategies and data to intelligent devices, robots and conveyor systems for the production of products.

### 4.2. Configuration Options and Operational Constraints

The CPPM production line is intended to produce three distinct types of goods: PA, PB, and PC. They are indicated as customised Bluetooth self-timer gift boxes, customised USB flash drive gift boxes and customised wood carving gift boxes. The range of products that can be customised includes gift box colours, product colours, engraving patterns, etc. The customer can personalise the processing features by selecting the personalisation. The production line automatically assembles the boxes for the above three types of product gift boxes without intervention. When an order is inserted urgently, the production line system can quickly implement a change of box validation of the validation model and reconfigure the scheduling strategy and production mechanism. This article abstracts the processing of the three types of products at the model level. Each product consists of five variants, each of which requires a number of distinct processes to complete. The product variants are distinguished by the numbers printed in the lower corner, for example, product PB for the two variants of product Pb_1_ and Pb_2_. The process that each version goes through during production will differ depending on the order. The number of versions has therefore been fixed at two for the subsequent convenience of performance statistics and analysis. The actual equipment resources that can provide processing capacity are denoted by R. Some machines can offer different processing capacities. For example, CNC Station offers the ability to engrave and drill, and denotes the operational processes offered as P_3_O_1_ and P_3_O_2_, as shown in Table 1. The “→” in the table indicates the process to be carried out for a particular version of the product. The process resource P_3_O_1_* provided by device R_4_ indicates that it is the same as the process provided by device R_3_ and can be used as an alternative to P_3_O_1_, as indicated by “⇨”. Pa_1_ versions can be produced with either R_3_ or R_4_ for the P_3_O_1_ process, depending on resource occupation. A blank space in the table indicates that the product is not processed during its passage through the process.

In addition, the product production process is constrained by the following process sequence constraints:

1. Process P_1_O_1_ is to be completed before process P_7_O_1_, and P_1_O_1_ must be the first process.
2. Processes P_6_O_3_ and P_7_O_1_ for the same product are mutually exclusive.
3. Processes P_7_O_1_, P_8_O_1_, and P_9_O_1_ must be completed in sequence, and process P_9_O_1_ must be the last process.
4. When a product is processed in several processes on the same equipment, the sequence is P_X_O_Y_ >> P_X_O_Y+Z_ (Z ≥ 1).

Meanwhile, to reduce the impact of the actual scheduling system on the accuracy of the performance analysis, a centralised first-in-first-out dynamic scheduling approach was used for modelling. The scheduling plan does not change when the resources do not change.

## 5. Model Development

The model to be analysed and constructed in this paper must be able to reflect the real-time availability of production line resources. To meet this requirement, this section proposes a modelling mechanism based on AutomationML. As the uniform communication interface for resource adaptation, the OPC UA protocol is used.

### 5.1. Construction of a Domain Formal Metamodel

Production line models are divided into metamodels and domain models according to their level of abstraction. The production line metamodel depicts the key modelling elements in the production line, i.e., the relationships between the modelling elements and their concepts. The construction of the metamodel is based on the given syntactic definition, which abstractly describes all of the production line domain’s elemental concepts. The domain model’s concepts and inter-concept relationships are clarified through the development of a metamodel. The metamodels are then instantiated to generate reusable domain model templates for various types of manufacturing domain models, thereby forming a model library. On this basis, the models in the model library are instantiated for specific application scenarios. Finally, the construction of the CPPM production line domain formal model is completed by model transformation and fusion.

**Figure 4:**
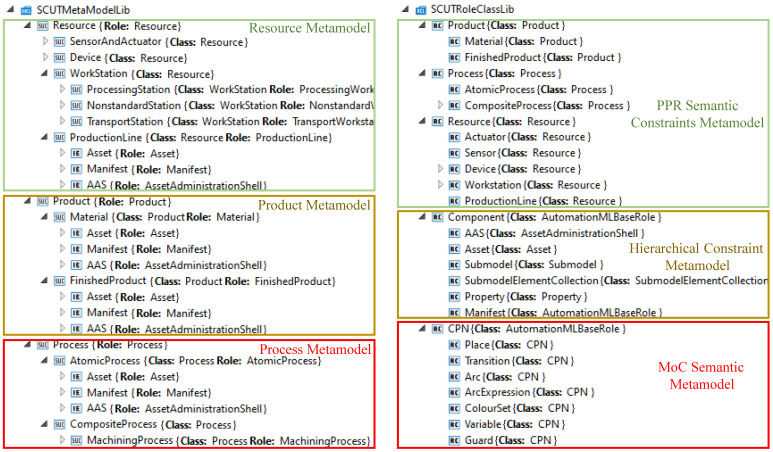
Metamodel of FDML in AutomationML notation.

The rational analysis and construction of the domain model elements’ relationships are essential for building a metamodel. Figure 4 illustrates the AutomationML representation of the system component metamodel and semantic metamodel. The development of a metamodel of production line components makes manufacturing domain knowledge visible and unifies developers’ understanding of production line systems. Duplication of work was avoided by enabling the categorisation and reuse of models. Modelling using a hierarchical approach balances model stability and consistency, makes each layer feature independent and simplifies complex system details.

### 5.2. Construction of a Domain Formal Model

**Figure 5:**
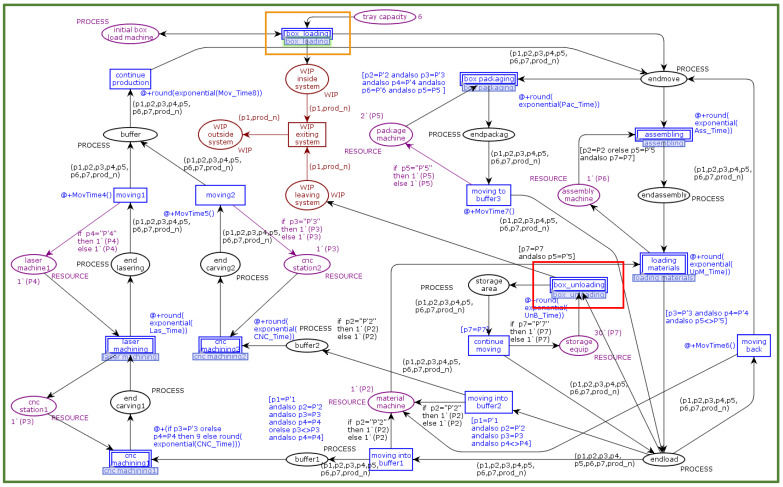
A snapshot of scenario “CPPM production-line” modelled on CPN tools.

**Figure 6:**
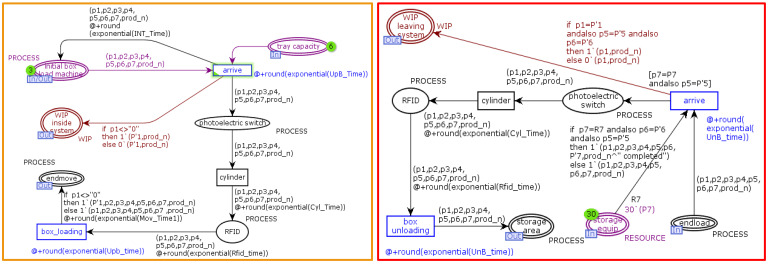
A snapshot of scenario “workstation layer” modelled on CPN tools.

Figure 5 shows a formal representation in CPN tools of a CPPM production line component domain model containing a production line layer and a workstation layer. A formal model of the workstation layer is shown in Figure 6, where the green tokens indicate the real-time available resources of the production line. The number contained within the token represents the processing capacity provided by the available resources. The layered modelling approach provides the ability to add or remove activity or sub-activity details from any process after the model development phase.

The production of the product begins with the “initial box load machine,” a token containing information about the ordered product and the to-be-processed procedure. Each process contains a hierarchical transition interface at the production line level. The token representing the product will pass through this hierarchical transition interface to the next process layer (the workstation layer) for processing, i.e., the processing and recording of data. The product token is then returned via the hierarchical place interface to the production line level to continue the production process. Each time a product token reaches a process node, it will be determined whether or not to process it based on scheduling rules, thereby meeting the production path’s flexibility requirements. In a simulation, if the order is successfully completed, all products will end up in the “storage area” with the suffix “completed”. For subsequent performance analysis, CPN tools monitoring tools are utilised. The “Data Coll” monitor in CPN tools is used to extract numerical data on the cycle time associated with the transition during the simulation in order to verify the position of the markers when the change takes place. The “Mark Size” monitor is applied to the “WIP inside the system” and “storage area” locations to extract location-specific production line data. Product quantity data such as “WIP” and “throughput” quantitative data such as average cycle time, throughput, and WIP are generated by the simulation of CPN Tools. The “Breakpoint” monitor is used to determine the simulation’s stopping conditions. The simulation stops when a specified feature arrives, such as the arrival of a specified machining completion state.

## 6. Simulations Results and Analysis

The performance of the CPPM production line is dependent on a variety of input factors, including the supply and transport of products and pallets, the execution of the process, and the dependability of resources. In this article, the product interval time (PIT) is used as a function variable for product supply. The transport factor uses the tray capacity (TC, number) and the conveyor speed (CS, m/min) as a function variable. Two key processes were used as execution factors: CNC-workstation processing time (CPT, minutes) and dual-arm robot processing time (DRT, minutes). The impact of the failure rate (FR, Permillage) on the makespan is used as a factor for resource reliability. This paper investigates the effect of each input determinant on average cycle time (average minutes), throughput (average number of products/hours), and work in progress (WIP) (products number).

The different levels of all input factors are shown in Table 2. Time dependent on the occurrence of the event, such as the supply and transport of products and pallets and the execution of processes in the model, are modelled using an exponential distribution function. Considering that any equipment failure affects the production line’s efficiency, each state transfer of a product on the line includes an additional processing probability function. The FR function indicates that the processing of the resource has a certain probability of failure. Due to the cyclic nature of the line, the current processing status of the product will be maintained in the event of a process failure. The product will then be returned to the failed position to continue processing until all processes have been completed.

**Figure 7:**
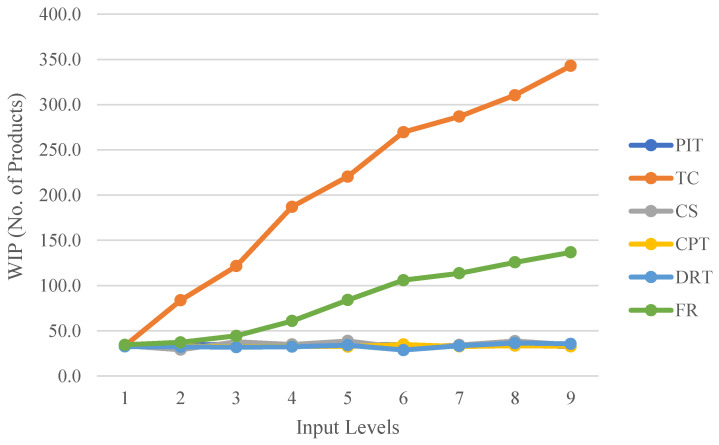
Variation in WIP at different input factor levels.

**Figure 8:**
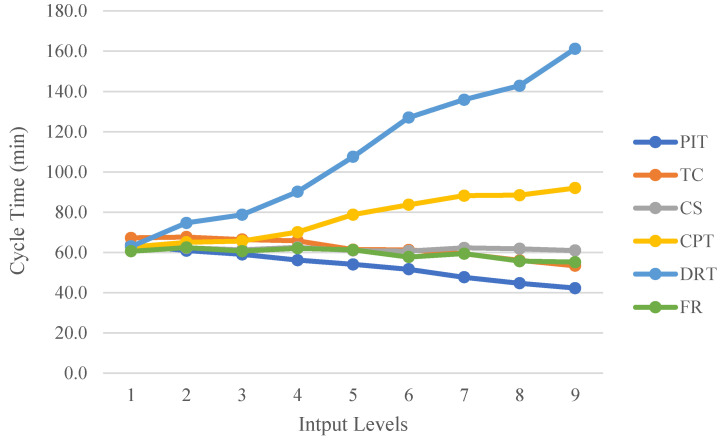
Variation in cycle time at different input factors levels.

**Figure 9:**
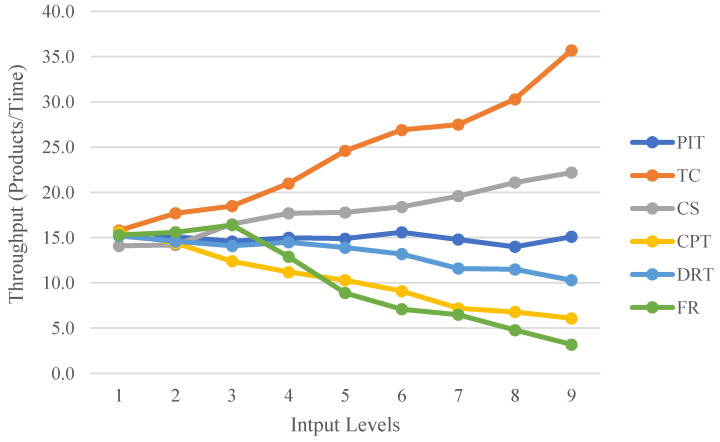
Variation in throughput at different input factors levels.

As shown in Table 2, these factors are also the part of the actual production line where the parameters can be adjusted. The time input values here are derived from the empirical statistical time range of the actual process, rather than the exponential function distribution that is usually used in the sense of KPI forecasting. The adjustable range of the parameters is therefore set to nine levels ranging from small to large. The simulation experiment was performed ten times for each set of parameters, assuming the same order. According to the One Factor at a Time Strategy (OFAT), only a single factor affecting the response is considered at a time, and all other factors are fixed at their respective levels for the simulation of the model. The CPN tool monitors the performance metric data generated by each set of input factors and records simulation results of the impact of each set of input factors on performance metrics, including average cycle time, throughput, and WIP, as depicted in Figure 7, Figure 8 and Figure 9.

The effects of the six input factors on WIP are shown in Figure 7. Among all the input data, TC and FR are regarded as the most influential factors in modifying the WIP. When both TC and FR increase, WIP also increases. The increase in WIP causes more semi-finished products to be kept on the production line, which increases the production cycle time for each product and is contrary to what the market expects. The impact of FR on WIP increases significantly after Level 2. The FR here is present for every device. Therefore, maintaining the equipment’s reliability within specified limits will significantly improve production performance indicators. Several additional factors have a negligible effect on the WIP and do not contribute to significant fluctuations in the WIP.

Figure 8 shows the effect of the six input factors on the mean cycle time. The effects of FR and CS on the mean cycle time are minimal. The input factor PIT, which reflects product supply, slowly decreases the average cycle time as the input level rises. An increase in TC also causes a reduction in the mean cycle time, but this effect is not significant until Level 4. An increase in TC also results in an increase in the number of WIPs; thus, it is crucial not to set TC too low or too high. As the CPT and DRT levels increase, so do the average cycle time and the time required to manufacture the product. However, DRT has a greater impact on the average cycle time than CPT. This is due to the fact that the dual-armed robot offers the widest range of process time adjustments of any process in the line and performs three processes, including packaging.

The impact of the six input factors on throughput is shown in Figure 9. The PIT has little effect on throughput because the majority of other processes require significantly more time than the PIT, which is adjustable time. Increasing CS and TC will increase yields, especially increasing TC. However, an increase in TC also leads to an increase in WIP, which increases production costs. Therefore, it is necessary to locate the appropriate number of pallets. An increase in CPT and DRT lengthens cycle time and consequently reduces throughput, resulting in a reduction in throughput. However, the magnitude of the impact of CPT and DRT on throughput differs, with CPT having a significantly greater effect. This is due to the fact that CNC-workstation 1 performs both processes, including drilling and engraving, whereas CNC-workstation 2, which complements the process capability, offers only engraving processes. FR can severely reduce throughput after Level 2, and the stability of the device is important in all cases.

The above analysis leads to the following production line design conclusions. The input parameters are optimised so that the average cycle time and WIP are minimised, and the throughput is maximised. The effect of PIT on WIP and throughput performance in production is negligible. However, an increase in PIT within a certain range will decrease cycle times; thus, the loading speed can be appropriately slowed down within a variable PIT range. Among the process input variables, a rise in DRT lengthens the cycle time. When designing the line, it is possible to include a packaging robot to share the processing time of the two-armed robot. For the other process execution input factor, CPT, the CNC-workstation 2 needs to be modified in subsequent production, e.g., by adding an automatic tool change system to increase the machining capacity of the CNC-workstation 2 in order to improve productivity. TC and CS are input variables to the production line’s transport factor, and both affect production performance. Lifting the CS will increase throughput to some degree; thus, whenever possible, production should maintain a rapid conveyor speed. The influence of TC on these performance indicators is a more complex input factor. Given that an increase in TC increases production costs (WIP and Smart Pallet), it is preferable to maximise TC within budget constraints. Finally, the resource reliability factor FR is one of the most important input factors. The equipment should be well maintained throughout the production cycle to ensure that it is as reliable and stable as possible in order to improve production efficiency.

**Figure 10:**
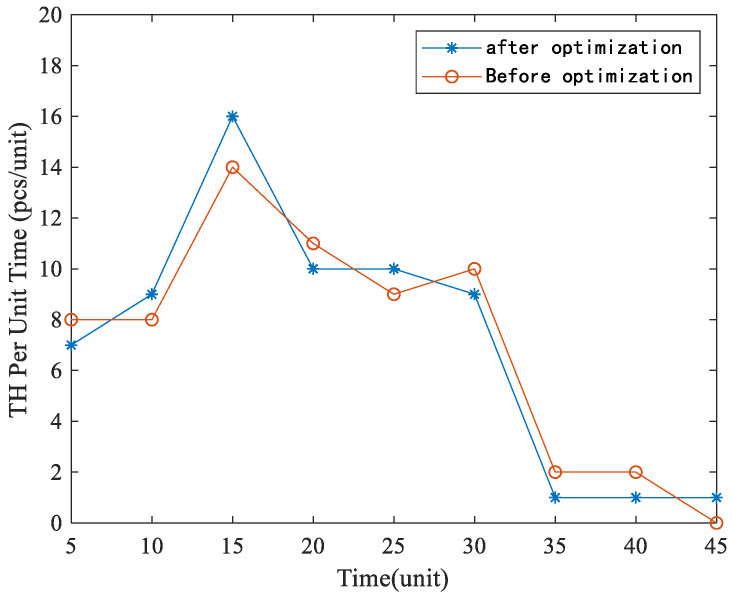
Comparison of experimental results before and after input parameter optimisation.

In order to validate the proposed method in this paper as an optimisation guide for the actual production line operation process, a set of random order production and simulation prediction experiments was designed for two consecutive rounds of throughput. Figure 10 illustrates the comparison of unit throughput metrics. By saturating the order and putting it into production, the total sum throughput deviation rate for the first 20 min is 4.76%. The production line reaches a maximum throughput at 10–15 min. The instantaneous maximum processing capacity of the production line on the experimental platform was increased from 14 to 16 ordered products, an increase of 14.3%. The experimental results demonstrate that the performance analysis method proposed in this paper has a definite improvement on the production line performance metrics.

## 7. Conclusions

This work has mainly focused on the investigation of modelling for performance analysis of production lines for plug-and-play manufacturing systems and can serve as a reference for similar approaches to formal verification and code generation based on a model-driven framework. A framework for modelling and performance analysis of customised plug-and-play production lines for CPPSs based on the AutomationML language for the factory domain is proposed. This method is based on a “multi-perspective, multi-hierarchy” description of the system components to build a smart manufacturing factory domain model, which is then converted into a formal model of the domain. The formalism of coloured Petri nets was used to construct a complete and compact model of the CPPM production line capable of storing real-time data on resource availability. A model of a personalised production system containing order data can be modelled, simulated, and analysed in a targeted way. The proposed theoretical framework is applicable to other complex CPPM performance evaluations and parameter optimisations. The results of the model simulation analysis indicate the need to keep PIT and FR as low as possible, using WIP, cycle time and throughput as performance indicators. The TC and CS variables are kept at the highest possible level, and bottleneck resources are kept more than twice as redundant as possible. Future research will focus on the following: further performance evaluation and optimisation of the various input parameters; the impact of faults and maintenance on productivity; and the quantitative study of the impact of the framework proposed in this paper on production performance.

## Figures and Tables

**Table 1:** Schematic of process planning for products.

Products	Type	R_1_	R_2_		R_3_		R_4_	R_5_	R_6_			R_7_	R_8_	R_9_
		P_1_O_1_	P_2_O_1_	P_2_O_2_	P_3_O_1_	P_3_O_2_	P_3_O_1_*	P_5_O_1_	P_6_O_1_	P_6_O_2_	P_6_O_3_	P_7_O_1_	P_8_O_1_	P_9_O_1_
PA	Pa_1_	→	→		→		⇨		→		→		→	→
	Pa_2_	→	→			→					→		→	→
PB	Pb_1_	→	→	→								→	→	→
	Pb_2_	→	→	→				→				→	→	→
PC	Pc_1_	→								→	→		→	→
	Pc_2_	→						→		→	→		→	→

**Table 2:** Different levels of various input basic factors.

Input Factors	Level 1	Level 2	Level 3	Level 4	Level 5	Level 6	Level 7	Level 8	Level 9
PIT	4	6	8	10	12	14	16	18	20
TC	6	9	12	15	18	21	24	27	30
CS	6.8	7.6	8.4	9.2	10	10.8	11.6	12.4	13.2
CPT	1.2	1.3	1.4	1.5	1.6	1.7	1.8	1.9	2
DRT	1	1.25	1.5	1.75	2	2.25	2.5	2.75	3
FR	0.001	0.03	0.06	0.09	0.12	0.15	0.18	0.21	0.24

## Data Availability

Not applicable.
